# Interaction of Avapritinib with Congo Red in Pancreatic Cancer Cells: Molecular Modeling and Biophysical Studies

**DOI:** 10.3390/ijms26051980

**Published:** 2025-02-25

**Authors:** Małgorzata Lasota, Daniel Jankowski, Anna Wiśniewska, Łukasz Szeleszczuk, Anna Misterka-Kozaka, Marta Kaczor-Kamińska, Marta Zarzycka, Maksym Patena, Tomasz Brzozowski

**Affiliations:** 1 Center for Biomedicine and Interdisciplinary Sciences, Faculty of Medicine, Jagiellonian University Medical College, 16 Grzegórzecka Street, 31-531 Krakow, Poland; anna.misterka@uj.edu.pl; 2 SSG of Targeted Therapy and Supramolecular Systems, Jagiellonian University Medical College, 16 Grzegórzecka Street, 31-531 Krakow, Poland; daniel.jankowski@student.uj.edu.pl (D.J.); maksym.patena@student.uj.edu.pl (M.P.); 3 Chair of Pharmacology, Faculty of Medicine, Jagiellonian University Medical College, 16 Grzegórzecka Street, 31-531 Krakow, Poland; anna.niepsuj@uj.edu.pl; 4 Department of Organic and Physical Chemistry, Faculty of Pharmacy, Medical University of Warsaw, 1 Banacha Street, 02-097 Warsaw, Poland; lszeleszczuk@wum.edu.pl; 5 Chair of Medical Biochemistry, Jagiellonian University Medical College, 7 Kopernika Street, 31-034 Krakow, Poland; marta.b.kaczor@uj.edu.pl (M.K.-K.); marta.zarzycka@uj.edu.pl (M.Z.); 6 Department of Physiology, Faculty of Medicine, Jagiellonian University Medical College, 16 Grzegórzecka Street, 31-531 Krakow, Poland

**Keywords:** avapritinib (BLU-258), Congo red, pancreatic cancer, cell viability, migration, tumor growth, apoptosis, targeted therapy

## Abstract

Pancreatic cancer is a malignant tumor with one of the worst prognoses among solid tumors, characterized by resistance to treatment. Therefore, there is an urgent need for new methods of targeted therapy. Previous studies have shown that the overexpression of receptor tyrosine kinases such as c-KIT or PDGFR can increase proliferation, migration, and invasion of cancer cells. The aim of our study was to analyze aggregates between a supramolecular carrier (Congo red, CR) and a tyrosine kinase inhibitor (BLU-258) as well as to investigate the effect of the free inhibitor and its aggregate with Congo red (CR-BLU-258) on selected properties of pancreatic cells, including these cells’ viability and three-dimensional cell spheroid cultures. To better understand the interactions between Congo red and BLU-258, we used molecular modeling in addition to biophysical methods. These attempts allowed us to determine the optimal molar ratio, which we used for in vitro studies on pancreatic cancer cell lines. A significantly greater decrease in the viability of the tested 3D cultures was observed after 48 h of incubation with CR-BLU-258, which resulted in a lower IC_50_ value for the tested co-aggregate compared with BLU-258 alone. Moreover, a higher resistance of PANC-1 and BxPC3 spheroid cells to the tested compounds was noted compared with the 2D culture model. A significantly lower response was observed in 3D cell cultures (BxPC3 and PANC-1) treated with BLU-258 alone compared with the 2D culture. Thus, our results showed that both BLU-258 (alone) and in its co-aggregate with Congo red exhibit anticancer activity, inhibiting the growth of pancreatic cancer cells and reducing their viability, survival, and migration. Both tested compounds also affected the phosphorylation of the selected signaling proteins. We conclude that the selected tyrosine kinase inhibitor (alone) and in its co-aggregate with Congo red exhibit anticancer activity and should be considered as a novel effective therapy against pancreatic cancer.

## 1. Introduction

Pancreatic cancer (PC), considered to be a disease with an extremely poor prognosis, remains largely refractory to available drug treatments [1]. By histopathological assessment, PC occurs in two main types of cancer: the exocrine type, which originates from cells of the exocrine pancreas, and the endocrine type, which originates from hormone-producing endocrine cells [2]. Exocrine tumors account for 95% of pancreatic cancer, and the most common pathological type is pancreatic ductal adenocarcinoma (PDAC) [3]. In cases where pancreatic cancer is diagnosed, only up to 20% of patients can undergo surgical treatment, which is the only possible curative therapy [4,5,6]. Despite advances in diagnostic tools, chemotherapy, and surgical treatment, the 5-year survival rate averages only 11.5% [5].

Unfortunately, the incidence of pancreatic cancer is increasing by 0.5% to 1.0% per year, and it is estimated to become the second-leading cause of cancer mortality by 2030 [7]. Most patients present with advanced, inoperable disease, and it is predicted that only about 12% will survive beyond 5 years [8]. In most cases, pancreatic cancer develops asymptomatically, which makes diagnosis difficult. Furthermore, symptoms are often nonspecific, and most patients report pain and nausea. In addition, these patients experience weight loss and loss of appetite. For this reason, most cases are diagnosed in an advanced stage of the disease [9].

Risk factors for pancreatic cancer can be divided into two groups: nonmodifiable factors, such as hereditary factors and age, and modifiable factors, such as tobacco exposure, alcohol consumption, diet, obesity, diabetes mellitus, as well as infections and certain abdominal surgeries [9,10].

Previous studies revealed that overexpression of receptor tyrosine kinases such as c-KIT and platelet-derived growth factor receptor (PDGFR), which show a very high structural similarity and almost identical mechanisms of action, may play an important role in the development of this cancer [11,12,13]. To date, the stem cell factor/c-KIT receptor pathway has been shown to enhance pancreatic cancer cell proliferation and invasion [11]. In addition, patients with concomitant abnormal expression of p53 and high levels of PDGFR-β and PDGFR expression present a higher risk of hematogenous metastases and a significantly poorer prognosis after surgery [13]. For these reasons, tyrosine kinase inhibitors may play an important role in the anticancer therapy of pancreatic cancer.

Avapritinib (BLU-258, AV) is an orally available, selective inhibitor of c-KIT and PDGFRA activation loop mutations [14,15]. BLU-258 is approved for the treatment of adults with nonresectable or metastatic GIST with PDGFRA exon 18 mutations, including PDGFRA D842V mutations, in the United States [14,16]. Due to its efficacy, the clinical development of BLU-258 for the treatment of systemic mastocytosis and late-stage solid tumors is ongoing in several countries [14]. Previous studies have shown that BLU-258 at non-toxic concentrations can impair the function of the ATP-binding transporters (ABC) ABCB1 and ABCG2 *in vitro* and restore chemosensitivity in multidrug-resistant cancer cells overexpressing ABCB1 and ABCG2 [14,17]. Our studies have shown that BLU-258 possesses the ability to form co-aggregates with Congo red.

Congo red (CR) is a chemical compound from the azo dye group [18,19]. As studies have shown, this compound can form specific complexes with antibodies bound to the antigen [18,20]. In addition, CR can stabilize the immune complex and enhance antigen–antibody interactions [18,20]. Our studies have shown that the combination of selected tyrosine kinase inhibitors with Congo red may be a promising approach in targeted anticancer therapy, characterized by reduced cytotoxicity and increased drug delivery stability [21].

Therefore, in the present study, we analyzed the formation of co-aggregates between the supramolecular carrier CR and a tyrosine kinase inhibitor (BLU-258, AV) and determined the effect of this inhibitor alone and of its co-aggregate with CR on selected properties of two pancreatic cancer lines (PANC-1 and BxPC3).

Molecular modeling methods are commonly used to study the adsorption of molecules and the formation of nanoparticles. Since the structures formed are usually highly disordered, such calculations are in some cases the method of choice to obtain information on the structure and properties of such systems. In the present study, we used periodic calculations that allowed for the consideration of intermolecular forces and the periodicity of a representative amorphous unit cell.

Complementing these modeling methods with biophysical techniques, we used dynamic light scattering (DLS) and UV-Vis spectroscopy to verify the results of our calculations and to better understand the behavior of the tyrosine kinase inhibitor AV in combination with CR. After determining the optimal CR-AV molar ratios, we examined the effect of the tested compounds on pancreatic cancer cells.

## 2. Results

### 2.1. The Interaction Studies Between AV and CR Molecules

To assess how the formation of the -CR-AV nanoparticles affects the system’s structure and energy, initially the AV and CR models were examined.

**Figure 1:**
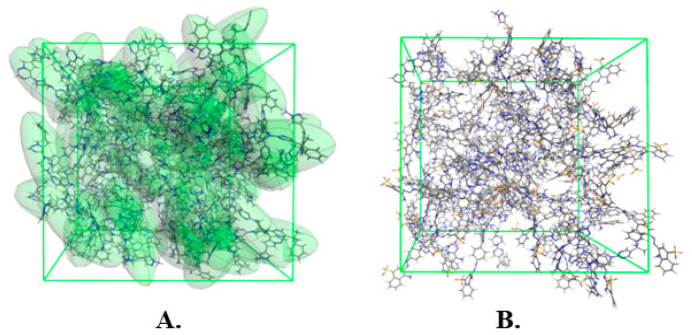
(**A**) Optimized amorphous unit cell of 100AV. Individual molecules are marked as green. (**B**) Optimized amorphous unit cell of 100CR.

The geometrically optimized amorphous unit cell of AV, containing 100 molecules, is presented in Figure 1A. The molecules of AV found inside this unit cell are randomly distributed and form no long-range ordered structures, proving the amorphous character of such system. The major intermolecular interactions in this system are H-bonds, with F and N atoms serving as donors and primary amine group serving as H-bond acceptor. The geometrically optimized amorphous unit cell of CR, containing 100 molecules, is presented in Figure 1B. As in the previous case, the molecules of CR, found inside this unit cell, are randomly distributed and form no long-range ordered structures, proving the amorphous character of the system. The major intermolecular interactions in this system are H-bonds, with O and N atoms serving as donors and the primary amine groups serving as H-bond acceptors. Additionally, the system is stabilized by the electrostatic interactions between the Na cations and CR anions.

**Figure 2:**
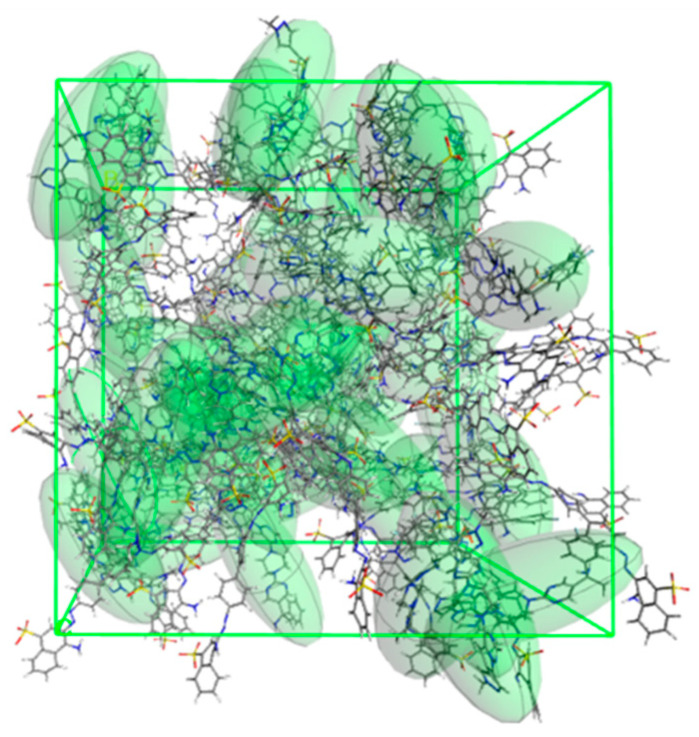
Optimized amorphous unit cell of the 50AV50CR system. Individual molecular ellipsoids of AV are marked as green.

Figure 2 presents the optimized amorphous unit cell containing 50 molecules of AV and CR each. Those molecules are evenly distributed, which indicates that the interactions between AV and CR are stronger than between two molecules of the same kind (AV-AV or CR-CR). All the interactions present in the homogenic structures, 100AV and 100CR, are also present in the 50AV50CR model. Furthermore, because of the formation of this heterogenic system, new types of H-bonds are also observed, with the -NH_2_ groups of both AV and CR serving as donors, while the O and N atoms of CR as well as the N and F atoms of AV serve as the H-bond acceptors.

**Figure 3:**
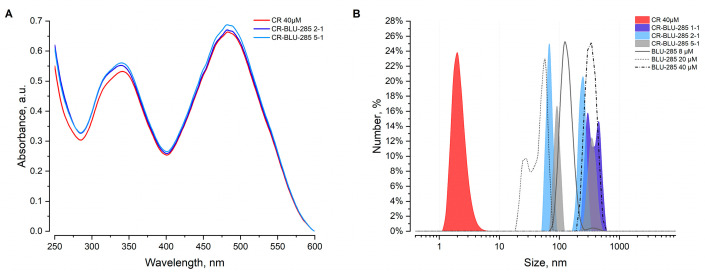
Analysis of aggregates formation. (**A**) UV-Vis spectra for selected CR-BLU-258 aggregate ratios and varying BLU-258 concentrations. (**B**) DLS measurements; the size distribution by number prevalence is depicted. The line profiles represent BLU-258 at different concentrations, while the filled curves represent aggregates of CR and BLU-258 at different ratios.

Table 1 presents the intermolecular interaction energies and volumes of the unit cells of the studied systems. For all three systems, the negative values of the intermolecular interaction energies (Eint) have been obtained. This indicates the stability of such systems and their tendency to form the nanoparticles. The more negative values of Eint found for A100 can explain the differences in the DLS results observed between CR 40 μM and AV 40 μM (Figure 3B). The AV tends to form the larger nanoparticles due to the higher absolute value of Eint. The value of Eint calculated for AV50CR50 was found to be between those for AV100 and CR100; however, the value of AV50CR50–0.5 (AV100 + CR100) was negative. This indicates that in solutions of equimolar concentrations of AV and CR, the heterogenous nanoparticles, containing both AV and CR, are preferable over the homogenic ones.

However, as the relative concentrations of AV and CR varies, the formation of nanoparticles with different than equimolar concentrations becomes more spontaneous. This agrees with the DLS results presented in Figure 3B; with the increase in the CR to AV concentration ratio, the two peaks observed in the DLS are more separated.

The calculated volume of AV100 is larger than that of CR100. This is also consistent with the DLS results, showing peaks for AV and CR at the same concentrations. The AV tends to form larger nanoparticles, not only because of the more negative Eint value, but also because the molar volume of AV is larger than that of CR. Similar to the Eint value, the molar volume of AV50CR50 is intermediate between those of AV100 and CR100.

### 2.2. Analysis of Nanoparticle Aggregates Formation

To investigate the effects of the interaction between CR and BLU-258, samples with different molar ratios were prepared and measured using dynamic light scattering (DLS) and UV–Visible (UV-Vis) spectroscopy.

UV-Vis measurements provide insight into the interactions primarily from the perspective of CR (Figure 3A). The absorption spectrum of CR displays a strong absorption band in the range of 450–550 nm. On the contrary, the maximum absorption of BLU-258 is observed near 250 nm [22]. Previous studies have demonstrated that a decrease in absorption within the 450–550 nm range indicates the formation of nascent supramolecular complexes involving CR and the ligands under examination [23].

In this study, the slight rise in absorption at 484 nm observed with increasing BLU-258 concentration suggests a different type of interaction between CR and BLU-258, influencing the spectral properties of CR in a novel manner compared with previously reported interactions [21]. DLS analysis indicated that BLU-258 forms aggregates in PBS with a monomodal and polymodal size distribution, showing an average hydrodynamic diameter ranging from 20 to 200 nm (Figure 3B). The CR alone at a concentration of 40 μM forms aggregates approximately 3 nm in size. The CR-BLU-258 aggregate profiles exhibit a bimodal distribution that is independent of the ratio.

In the case of the 2:1 ratio (excess CR), the first aggregate peak partially overlaps with the distribution peak of BLU-258 alone at that concentration, and the second peak represents larger particle sizes. In the case of the 5:1 ratio, we observe a bifurcation in the nanoparticle population, with lower and higher mean hydrodynamic sizes compared with BLU-258 alone at the same concentration (8 μM). These results indicate that the interaction between CR and BLU-258 leads to the formation of larger aggregates, suggesting significant molecular interactions resulting in a potential co-aggregation between CR and BLU-258.

### 2.3. Effect of BLU-258 and CR-BLU-258 on the Viability and Survival of Pancreatic Cancer Cells

**Figure 4:**
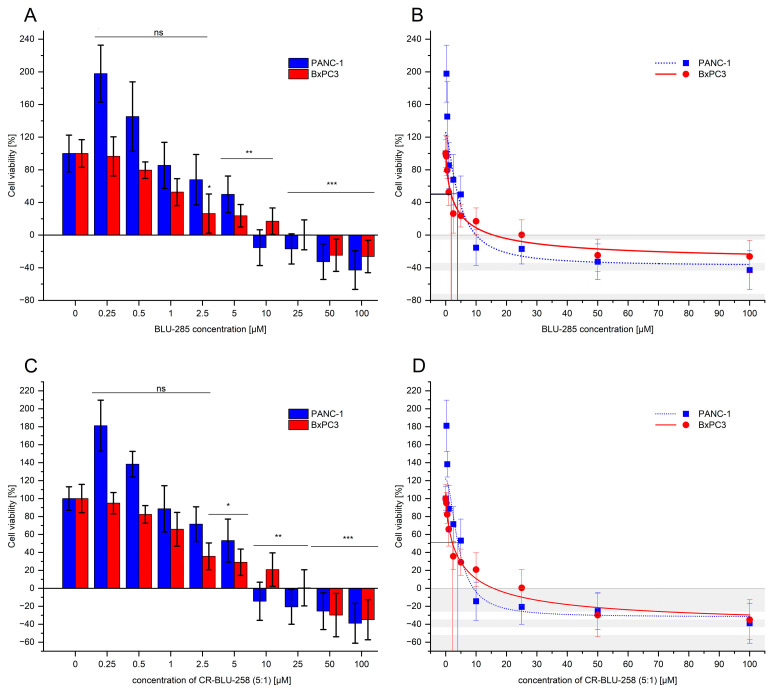
(**A**,**C**) The dose-dependent effect of BLU-258 (alone) and in aggregates with Congo red (CR-BLU-258) on the viability of PANC-1 and BxPC3 cells after 48 h incubation. Statistical significance between untreated and treated samples was evaluated using ANOVA with Dunnett’s post-test: ns, non-significant (*p* > 0.05) in comparison with a control sample (without BLU-258, with 0.1% DMSO); * 0.01 < *p* < 0.05; ** 0.001 < *p* < 0.01; *** *p* < 0.001. Growth inhibition curve in standard culture conditions (**B**,**D**).

To determine the effect of BLU-258 and its aggregates with CR (CR-BLU-258) on pancreatic cancer cell lines (PANC-1 and BxPC-3), MTS assays were performed for up to 72 h. As shown in Figure 4A,C, the exposure of PANC-1 and BxPC3 cells to BLU-258 alone and CR-BLU-258 resulted in significant dose-dependent suppression of the proliferation of pancreatic cancer cells compared with the control cultures (i.e., without the investigated compounds and with 0.1% DMSO). To fit the sigmoidal dose–response curve model obtained for BLU-258 alone and its aggregates with CR (CR-BLU-258), 50% and 90% inhibition of cell viability were established (Figure 4B,D and Table 2).

At concentrations higher than 5 µM of BLU-258, complete inhibition of PANC-1 cell viability was observed. A similar effect was obtained in BxPC3 cells at concentrations higher than 10 µM with the tested inhibitor. The absorbance value was lower than the value obtained for day 0, which may indicate a cytotoxic effect of BLU-258. 

A similar cytostatic effect was observed when BLU-258 was used with Congo red (CR-BLU-258) at the optimal molar ratio of 5:1 (Figure 4C); the statistical comparisons performed between BLU-258 and CR-BLU-258 did not show statistical significance (Student’s *t*-test, *p* > 0.05). However, the obtained IC_50_ and IC_90_ values were slightly higher than those for the inhibitor alone (the exception was the IC_90_ value for the PANC-1 line).

In addition, the MTS assay was used to determine the effect of Congo red alone on the viability of the tested pancreatic cancer lines (PANC-1 and BxPC3; Supplementary Figure S1). The results showed a much smaller effect of Congo red alone on the viability of the cell lines studied. The percentage of cell viability at the highest CR concentration of 100 µM was 74.9% (for the PANC-1 line) and 63.5% (for the BxPC3 line).

A similar effect of Congo red on viability was observed for the normal human gastric fibroblast cell line (Supplementary Figure S4A,B).

### 2.4. Effect of BLU-258 and CR-BLU-258 on Cell Cytotoxicity

**Figure 5:**
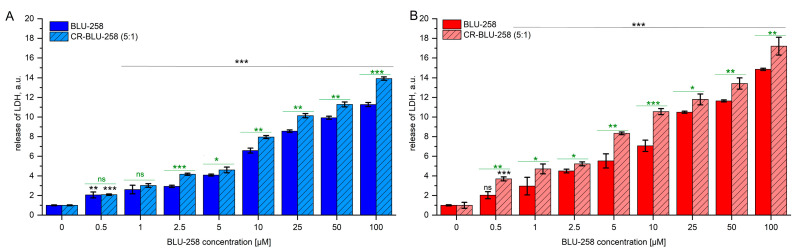
The cytotoxic effect of BLU-258 alone and in aggregates with Congo red (CR-BLU-258) on pancreatic cell lines ((**A**)—PANC-1; (**B**)—BxPC3) was assessed using LDH assay after 48 h incubation. Statistical significance between untreated and treated samples assessed using ANOVA with Dunnett’s post-test compared with the control sample (without BLU-258 or CR-BLU-258; with 0.1% DMSO)—black asterisks; statistical significance between BLU-258 and CR-BLU-258 assessed using Student’s *t*-test—green asterisks; ns—not significant (*p* > 0.05); * 0.01 < *p* < 0.05; ** 0.001 < *p* < 0.01; *** *p* < 0.001.

The cytotoxicity of the tested compounds on the tested pancreatic cancer cells was evaluated by LDH release after 48 h incubation with the tested compounds (Figure 5, Supplementary Figure S3). A dose-dependent increase in LDH release was observed with both BLU-258 and CR-BLU-258. At the highest concentration of BLU-258 tested, 100 µM, it was more than 11-fold higher for the PANC-1 cell line compared with control levels (Figure 5A) and over 14-fold higher for the BxPC3 cell line than for the control (Figure 5B). Furthermore, the CR-BLU-258 was observed to increase LDH secretion compared with avapritinib alone, and the observed differences between the single and combined treatments were statistically significant (*p* < 0.05) for the PANC-1 cell line at concentrations ≥ 2.5 µM and for the BxPC-3 cell line over the tested concentration range.

Moreover, while Congo red alone caused some LDH release after 48 h of incubation, it was significantly lower, approximately 10-fold lower, than that caused by BLU-258 alone or the co-aggregates tested (Supplementary Figure S3). A similar effect to Congo red was observed with normal human gastric fibroblasts (Supplementary Figure S4C).

### 2.5. Prolonged Treatment of Pancreatic Cells with BLU-258 or CR-BLU-258 Increases Both Apoptosis and Necrosis

**Figure 6:**
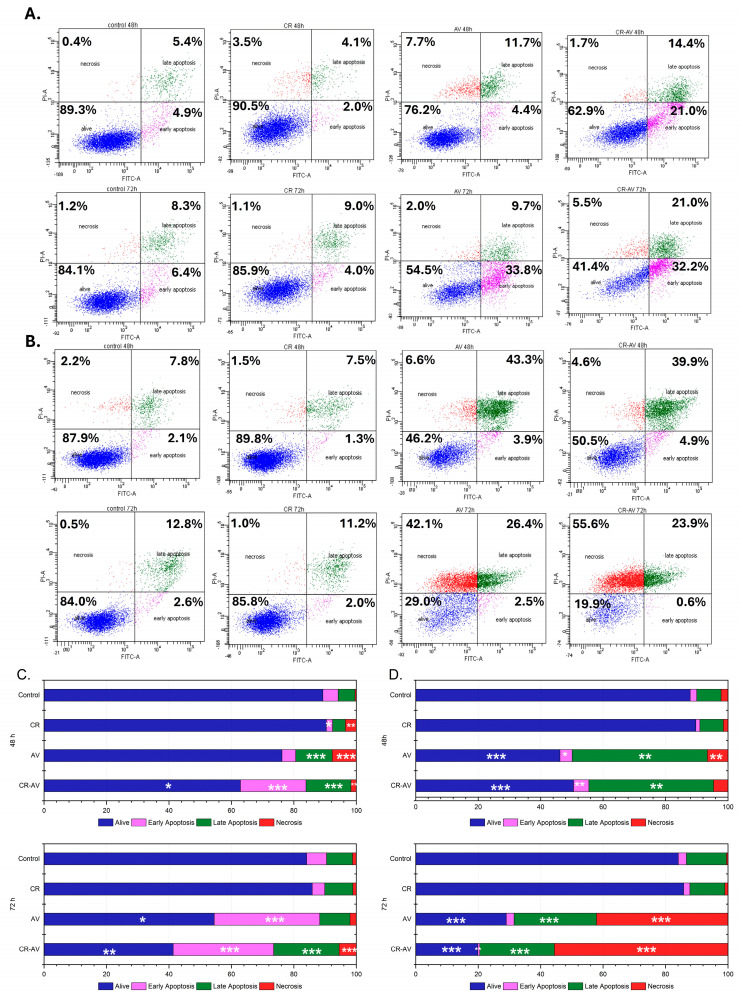
The effect of the BLU-258 inhibitor alone and in aggregates with Congo red (CR-BLU-258) on the apoptosis and necrosis of pancreatic cells. The images show flow cytometry analysis of Annexin V and PI staining presented on a dot plot graph. Graphic representation of four cell states: alive—the lower left square; cells undergoing necrosis—the upper left square; cells in early apoptosis—the right lower square; and cells in late apoptosis—the upper right square. PANC-1 (**A**) and BxPC-3 (**B**) cells were incubated with BLU-258 (AV) or CR-BLU-258 (CR-AV; at the IC50 concentrations). Cumulative bar charts show the interrelation between the state of cells after 48 h and 72 h exposure to BLU-258 or CR-BLU-258 on PANC-1 (**C**) and BxPC3 (**D**) cells. Statistical significance between the investigated samples was evaluated using the Mann–Whitney U test: * 0.01 < *p* < 0.05; ** 0.001 < *p* < 0.01; *** *p* < 0.001.

Next, we tested the effect of the inhibitor itself and the tested aggregate (at the IC_50_ concentration) on the survival of the pancreatic cancer cell lines using Annexin V and propidium iodide (Figure 6). We also tested the effect of Congo red alone (in the concentration range of 2.5–60 µM) on the survival of the selected cell lines (Supplementary Figure S2).

Our results showed a slight increase in the percentage of apoptotic cells after 48 h of incubation with BLU-258 (Figure 6A,C) compared with the control (untreated cells with 0.1% DMSO) for the PANC-1 line. A significant increase in apoptotic cells of the PANC-1 line was observed after extending the incubation time with the tested inhibitor to 72 h (Figure 6A,C). The treatment of these pancreatic cell lines with CR-BLU-258 caused a greater increase in the percentage of apoptotic cells than in the case of the inhibitor alone; the observed differences between single and combination treatments were statistically significant (*p* < 0.05). The results of our study also showed a significant increase in the percentage of apoptotic cells compared with the control group in the BxPC3 cells treated with BLU-258 (Figure 6B,D). After extending the incubation with the inhibitor to 72 h, a further decrease in the survival of tumor cells and an increase in the number of necrotic cells were observed.

Moreover, treatment of BxPC3 cells with the CR-BLU-258 aggregate (5:1 ratio) increased the percentage of apoptotic cells after 48 h of incubation and that of necrotic cells after 72 h compared with cells treated with the inhibitor alone, and the observed differences between the single and combination treatments were statistically significant (*p* < 0.05). Importantly, CR alone had no significant effect on the survival of both tested cancer cell lines, regardless of the incubation time. Regardless of the Congo red concentration, the percentage of apoptotic cells was low and in no case exceeded 12% (Supplementary Figure S2). Furthermore, the effect of Congo red on the survival of normal human gastric fibroblasts was examined (Supplementary Figure S4D,E). The obtained results showed that Congo red does not significantly affect the survival of HGF cells. Even at the highest concentrations used (60 µM, 100 µM), the percentage of apoptotic cells did not exceed 5%.

### 2.6. Effect of BLU-258 Monotherapy and Its Aggregate with CR on Pancreatic Cancer Cell Migration

**Figure 7:**
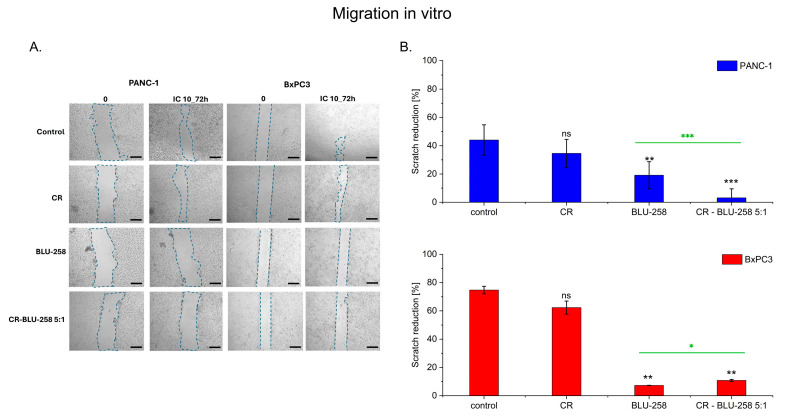
(**A**) The effect of BLU-258 and CR-BLU-258 on the motility of the pancreatic cell lines PANC-1 and BxPC3. Imaging under a 5× objective. Scale bar 400 µm. The wound area is marked with a dashed line. (**B**) Percentage of scratch reduction after 72 h (n = 4). Statistical significance between the non-treated and treated samples (black asterisks) was evaluated using the unpaired Student’s *t*-test: ns—non-significant (*p* > 0.05); * 0.01 < *p* < 0.05; ** 0.001 < *p* < 0.01; *** *p* < 0.001; statistical difference between BLU-258 and CR-BLU-258 (marked by green asterisks).

Pancreatic cancer cells (PANC-1 and BxPC3) were treated with Congo red, the inhibitor BLU-258, and the tested aggregate (CR–BLU ratio 5:1). Cancer cell migration was assessed using a wound healing/scratch assay (Figure 7). Optical images show an overgrowth of pancreatic cancer cells by scratches over time (Figure 7A). The photos were taken immediately after the wound as well as 72 h later with an appropriate control (untreated cells with 0.1% DMSO).

The obtained results showed a significant reduction in cell motility after exposure to both the inhibitor alone and CR-BLU-258 5:1 in the tested cell lines (Figure 7B).

Untreated cells and cells treated with CR alone had closed the wound almost completely within approximately 72 h, while the scratches in cells treated with BLU-258 and CR-BLU-258 5:1 remained open, regardless of cell type. These results indicate that after 72 h, the motility of PANC-1 and BxPC3 cell lines was significantly impaired by BLU-258 and CR-BLU-258 at the IC10 concentration. The observed effect on the inhibition of migration was similar in both cell lines tested.

### 2.7. Effect of BLU-258 and CR-BLU-258 on Selected Signal Transduction Mediator Proteins

Finally, the effect of the inhibitor BLU-258 and its aggregates with CR on selected signal transduction mediators was assessed.

**Figure 8:**
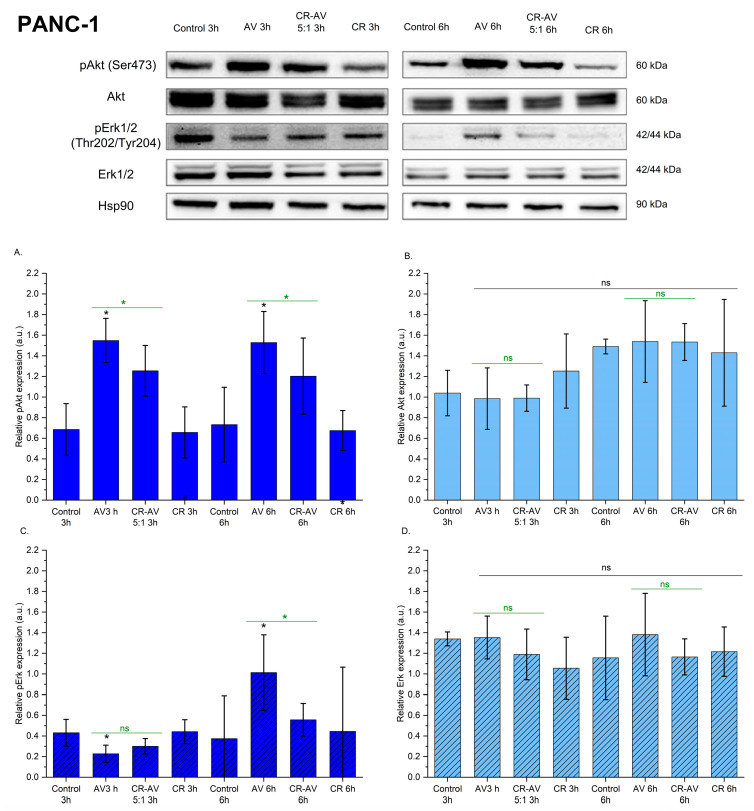
The effect of the investigated compound BLU-258 (AV) and its aggregate with Congo red (CR-BLU-258) on the p-AKT, AKT, p-ERK, and ERK protein levels in the PANC-1 cell line after 3 and 6 h of incubation with inhibitors (at the IC50 concentration). Results show representative Western blot images with immunodetection and densitometric evaluation of the bands (**A**–**D**). Statistical significance between untreated and treated samples evaluated using ANOVA with Dunnett’s post-test compared with the control sample (without BLU-258 or CR-BLU-258; with 0.1% DMSO)—black asterisks; statistical significance between BLU-258 and CR-BLU-258 assessed using Student’s *t*-test—green asterisks; ns—not significant (*p* > 0.05); * 0.01 < *p* < 0.05.

In the case of the PANC-1 cell lines, the phosphorylation of Akt at Ser473 was increased after incubation with BLU-258 and its aggregate with Congo red (CR-BLU-258) (Figure 8A). Treatment of PANC-1 cells with the tested compounds resulted in a decrease in the phosphorylation of pErk1/2 (Thr202/Tyr204) after 3 h of incubation (Figure 8C). When the incubation of PANC-1 cells with the tested compounds was extended to 6 h, an increase in the phosphorylation of Erk1/2 was observed compared with the control (untreated cells with 0.1% DMSO). No significant differences were observed between the treated and control PANC-1 cells in the total amount of Akt and Erk1/2 (Figure 8B,D).

**Figure 9:**
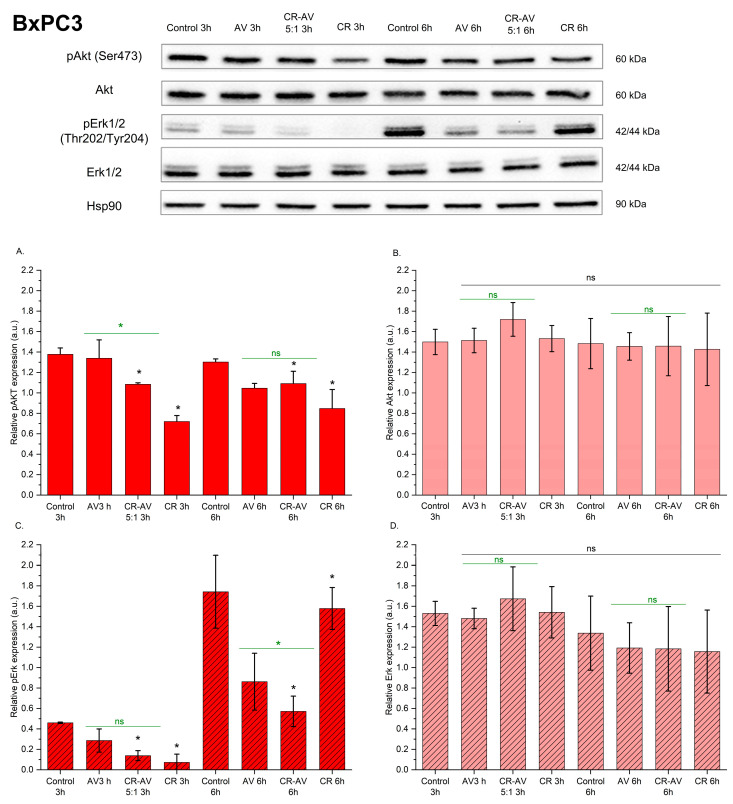
The effect of the investigated compound BLU-258 (AV) and its aggregate with Congo red (CR-BLU-258) on the p-AKT, AKT, p-ERK, and ERK protein levels in the BxPC3 cell line after 3 and 6 h of incubation with inhibitors (at the IC50 concentration). Results show representative Western blot images with immunodetection and densitometric evaluation of the bands (**A**–**D**). Statistical significance between untreated and treated samples evaluated using ANOVA with Dunnett’s post-test compared with the control sample (without BLU-258 or CR-BLU-258; with 0.1% DMSO)—black asterisks; statistical significance between BLU-258 and CR-BLU-258 assessed using Student’s *t*-test—green asterisks; ns—not significant (*p* > 0.05); * 0.01 < *p* < 0.05.

Treatment of BxPC3 cells with BLU-258 or CR-BLU-258 5:1 decreased Akt phosphorylation at Ser473 (Figure 9A). Furthermore, in the BxPC3 cell line, a decrease in the level of pErk/2 phosphorylation was also observed under the influence of the tested compounds compared with the untreated cells. Similarly to the PANC-1 line, in the BxPC3 line, no effect of the tested compounds on the total amount of Akt and Erk1/2 was observed.

### 2.8. Effect of BLU-258 and CR-BLU-258 on 3D Cell Cultures

In addition, we determined the effect of BLU-258 and its aggregates with CR (CR-BLU-258) on 3D cell cultures.

**Figure 10:**
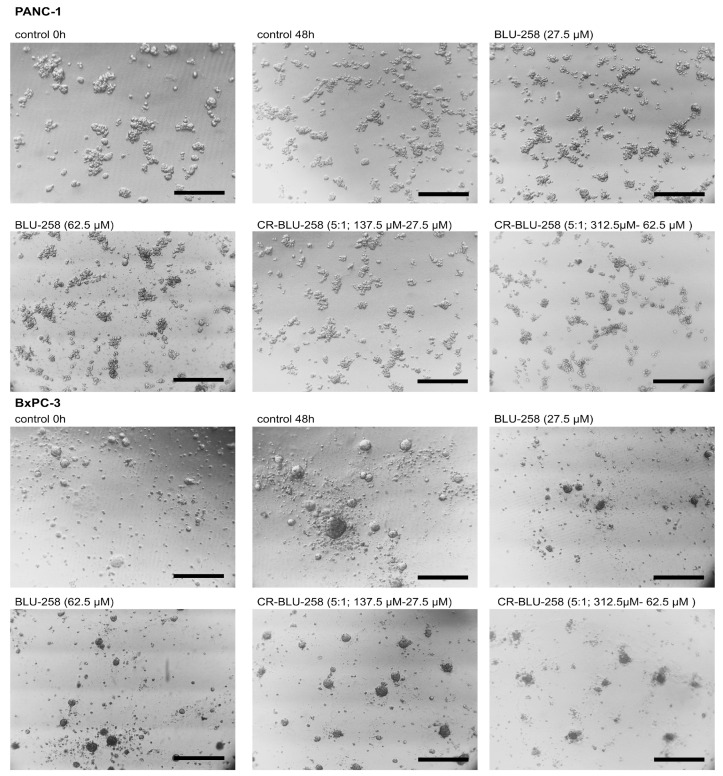
Morphology of the 3D cell cultures of PANC-1 cells and BxPC3 cells incubated for 48 h with BLU-258 or CR-BLU-258 and without the tested compounds (control with 0.1% DMSO); scale bar = 200 μm.

The morphology of 3D cell cultures under the influence of the tested compounds was monitored using a microscope (Figure 10). We observed a concentration-dependent disintegration of 3D structures under the influence of BLU-258 and CR-BLU-258.

To determine the effect of BLU-258 and its aggregates with CR (CR-BLU-258) on 3D cell cultures of pancreatic cells (PANC-1 and BxPC-3), the CellTiter-Blue^®^ Cell Viability Assay was performed.

**Figure 11:**
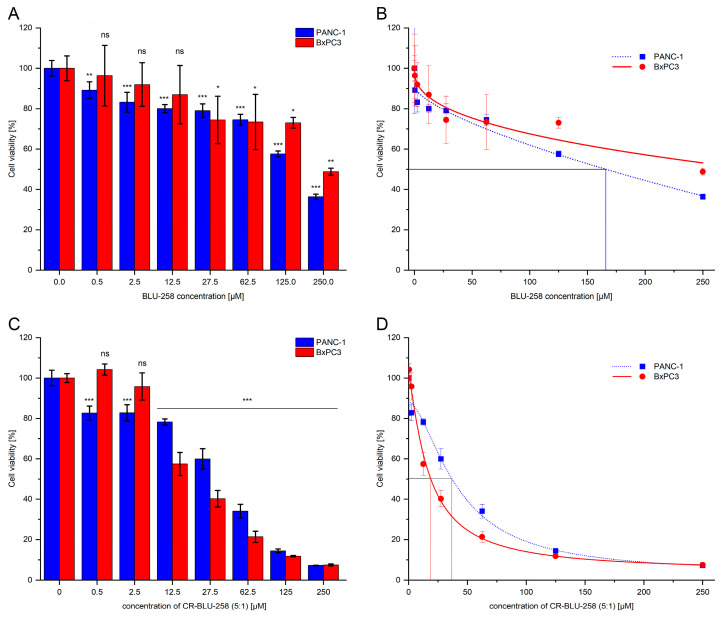
The dose-dependent effect of BLU-258 alone and in aggregates with Congo red (CR-BLU-258) on the viability of 3D PANC-1 and 3D BxPC3 cells after 48 h incubation (**A**,**C**). Statistical significance between untreated and treated samples was evaluated using ANOVA with Dunnett’s post-test: ns—non-significant (*p* > 0.05) in comparison with a control sample (without BLU-258, with 0.1% DMSO); * 0.01 < *p* < 0.05; ** 0.001 < *p* < 0.01; *** *p* < 0.001. Growth inhibition curve in standard culture conditions (**B**,**D**).

The 10% and 50% inhibition of the cell viability was determined by fitting a sigmoidal model of the dose-dependent curve obtained for BLU-258 alone and its aggregates with CR (CR-BLU-258) (Figure 11B,D and Table 3).

These results confirmed a higher resistance of PANC-1 and BxPC3 spheroid cells to the tested compounds compared with the 2D culture model. A significantly lower response was observed in 3D cell cultures (BxPC3 and PANC-1) treated with BLU-258 alone compared with the 2D culture (Figure 4 and Figure 11). A significantly greater decrease in the viability of the tested 3D cultures was observed after 48 h of incubation with CR-BLU-258, also resulting in a lower IC_50_ value for the tested co-aggregate compared with BLU-258 alone (Figure 11, Table 3). The statistical comparisons performed between BLU-258 and CR-BLU-258 showed statistically significant differences at concentrations ≥ 27.5 µM for the PANC-1 cell line and ≥12.5 µM for the BXPC-3 cell line (Student’s *t*-test, *p* < 0.05; Supplementary Figure S5).

## 3. Discussion

Pancreatic cancer accounts for 1.8% of all cancers [24,25]. Despite rapid advances in modern medical technology, pancreatic cancer remains a cancer with a low 5-year survival rate, and it is difficult to detect early [24]. At the time of diagnosis, pancreatic cancer is often in an advanced stage and frequently spreads to other organs of the body [24,26].

With the introduction of new surgical techniques and medical therapies, such as laparoscopic techniques and neoadjuvant chemotherapy, the treatment of pancreatic cancer continues to evolve, but with only modest improvements in treatment outcomes [4,26,27]. Furthermore, most tumors cannot be removed by surgery, as they metastasize and invade large vessels behind the pancreas [26]. In such cases, palliative treatment or cytotoxic chemotherapy is most often used [4,27]. Therefore, there is still a need to introduce new compounds that could play an important role in pharmacotherapy.

Previous studies showed that mutations in the c-KIT or platelet-derived growth factor receptor (PDGFR) drive ligand-independent constitutive kinase activity and downstream signaling, resulting in increased proliferation, migration, and survival of pancreatic cancer cells [11,28,29,30]. For these reasons, we attempted to investigate one of the receptor tyrosine kinase inhibitors, BLU-258 (AV).

BLU-258 is an ATP-competitive inhibitor that binds to the active conformation of PDGFRA and c-KIT kinases [14,15]. In vitro, BLU-258 disrupts c-KIT signaling by inhibiting its phosphorylation and activating signal transduction proteins such as AKT and STAT3 in human mast and leukemic cell lines [31,32,33]. Furthermore, this compound has shown efficacy against the PDGFRA-D842V mutation, which has been infamously insensitive to all known kinase inhibitors for over a decade [15,33]. BLU-258 is currently being studied in patients with inoperable, refractory solid tumors [33,34]. The latest studies from 2024 show how this drug binds to the receptor tyrosine kinases PDGFR and c-KIT, which may be of great importance in the design of derivatives of this compound as well as in determining key pharmacophoric features to overcome drug resistance and limit the potential penetration of the blood–brain barrier [15].

To improve the solubility of BLU-258, we also included in our studies an additional compound, Congo red (CR), which, as documented by our previous studies, can be used in potential targeted therapy. Previous studies have shown that CR has the ability to form complexes with doxorubicin (an anthracycline antibiotic with anticancer activity) [35,36], and it interacts with selected inhibitors of tyrosine kinase activity [21,37,38].

The results of our biophysical and molecular modeling provide an attempt to understand the interactions between CR and BLU-258, revealing the structural and energetic dynamics of their co-aggregated nanoparticles. UV-Vis spectroscopy data indicate that CR exhibits a strong absorption band in the range of 450–550 nm, and the increase in absorption at 484 nm at higher BLU-258 concentrations suggests a distinct interaction influencing the spectral properties of CR. The observed spectral changes imply that interactions between CR and BLU-258 could involve specific binding or aggregation mechanisms that alter the electronic environment of CR. Such changes are commonly observed during the formation of nanostructures and often serve as key indicators to monitor these processes [39].

The DLS analysis further elucidated the nature of these aggregates. Pure BLU-258 forms aggregates with a wide size distribution, whereas CR alone forms much smaller particles. The CR-BLU-258 complexes displayed a bimodal size distribution independent of the molar ratio, which points to the presence of interactions leading to the formation of distinct nanoparticle populations. Notably, at a 2:1 ratio of CR to BLU-258, the aggregates partially overlap with the BLU-258 distribution, suggesting the presence of mixed aggregates. At a 5:1 ratio, the bifurcation in the size distribution suggests the formation of two distinct populations of nanoparticles. This is likely driven by the excess CR, which facilitates different aggregation pathways, a phenomenon observed in studies of nanoparticle aggregation principles and modeling [40].

Molecular modeling provided additional insights into the structural and energetic properties of these aggregates. The negative intermolecular interaction energies (Eints) for all systems indicate their stability and propensity to form nanoparticles. The negative Eint for AV50CR50–0.5 (AV100 + CR100) underscores the preference for heterogeneous nanoparticle formation in equimolar solutions. This aligns with the DLS data showing distinct nanoparticle populations at varying CR-to-BLU-258 ratios, corroborating the notion that CR promotes the formation of stable, heterogeneous aggregates with BLU-258.

The obtained aggregates of CR with BLU-258 in a molar ratio of 5:1 were then used to assess the effect of this type of compound on human pancreatic cancer cell lines (PANC-1 and BxPC3). The conducted assay allowed us to compare the effect of BLU-258 alone with those of its co-aggregates with CR. Our studies have shown that the tested compounds, BLU-258 and CR-BLU-258, inhibited the growth of pancreatic cancer cell lines (PANC-1 and BxPC3) and significantly reduced the migration of cancer cells. The determined IC_50_ values in the 2D model for BLU-258 and CR-BLU-258 were comparable. Moreover, the CR-BLU-258 reduced the percentage of viable cancer cells (regardless of the tested cell line), showing a stronger proapoptotic or necrotic effect than the inhibitor alone; the observed differences between the single and combination treatments were statistically significant.

Interestingly, CR itself did not affect survival in the pancreatic cancer cell lines studied. A similar effect of CR alone on cancer cells has previously been observed in bladder cancer [21]. Furthermore, the compounds that we tested affected the phosphorylation level of the Akt and Erk1/2 proteins. Notably, we observed an increase in Akt phosphorylation in PANC-1 cells following treatment with CR-BLU-258 even though BLU-258, alone or in combination with CR, induced significant cell death. This finding may suggest a complex interplay between Akt signaling and the cellular response to treatment. However, we did not perform comprehensive phospho-proteomic analyses, such as unbiased phospho-site profiling or RPPA phosphorylation analysis, to obtain a broader picture of the signaling changes induced by avapritinib and the combination treatment. Interestingly, the observed increase in pAkt in the PANC-1 cells and its decrease in the BxPC3 cells could be attributed to differences in their epigenetic background and crosstalk in the signaling network. It is plausible that PANC-1 cells may exert compensatory or feedback mechanisms that lead to increased Akt activation in response to drug-induced stress, possibly as a survival strategy. In turn, the drug may effectively inhibit upstream signaling pathways in BxPC3 cells, leading to decreased levels of pAkt. The biological significance of this differential response lies in the distinct cellular contexts of these cell lines, highlighting potential differences in their dependence on the PI3K/Akt pathway, which is essential for survival. This finding suggests that the drug exerts context-dependent effects, indicating the importance of personalized approaches in the treatment of pancreatic cancer. However, we believe that future research can focus on and identify these underlying mechanisms to improve therapeutic strategies.

Recent studies have shown that 3D cell cultures are essential for assessing drug sensitivity in cancer models in vitro and enable the comparison of cancer cells’ responses to cytostatic drugs in vitro [41,42,43,44,45]. Interestingly, a greater resistance of 3D cell cultures to the tested compounds has been observed [41,42,43,44].

Herein, we present experimental evidence that 2D cell cultures showed increased sensitivity to both BLU-258 alone and CR-BLU-258 compared with 3D cell cultures, both in terms of cell viability and the concentration required to achieve 50% of growth inhibition.

Furthermore, our studies revealed a synergistic effect of the CR-BLU-258 complex, particularly in 3D cell cultures, where a significantly higher sensitivity was observed compared with treatment with the inhibitor alone. This synergy is likely due to the interactions between CR and BLU-258, which improves the bioavailability and efficacy of the inhibitor. Specifically, BLU-258 tends to form large aggregates, as shown by DLS measurements, limiting its solubility and availability for cellular uptake. By forming a complex with CR, these limitations are alleviated, potentially through increased stability, size distribution, and delivery in a 3D environment. Importantly, our studies also show that the IC_50_ value is significantly reduced by the tested CR-BLU-258 complex compared with the inhibitor alone.

The results obtained indicate a complex mechanism of action of the tested compounds, including both the inhibition of cells’ growth and their ability to spread. It is important to emphasize that the induction of apoptosis and inhibition of migration alone may not be sufficient indicators of therapeutic efficacy. However, in combination with the confirmed antiproliferative activity and modulation of signaling pathways, our findings suggest that the tested drugs possess therapeutic potential.

Moreover, the observed effects in 3D models additionally confirm these conclusions, because such models better predict the tumor responses to treatment in living organisms. Our results indicate that the tested compounds exhibit multifaceted anticancer activity, not only by inducing apoptosis and inhibiting migration but also by reducing cell viability and modulating key signaling pathways. The use of 3D models reinforces the significance of the data obtained, suggesting the potential drug’s efficacy in in vivo conditions.

However, we recognize the importance of further studies to better confirm the translational relevance of our findings.

It should be noted that the toxicity of CR recently triggered considerable attention [46]. Studies have shown that CR administered orally is mutagenic and is associated with the reduction of azo-nitrogens to amine groups by bacteria in the intestinal microflora, resulting in the formation of highly toxic benzidine [47,48,49]. However, CR injected intravenously binds with a high affinity to serum albumin, is then absorbed almost selectively by Kupffer cells, and is finally excreted into the bile [50,51]. Part of CR can also be absorbed by other cells of the reticuloendothelial system, such as macrophages of the spleen and lungs, or extracted by the kidneys into the urine [50].

The clinical use of Congo red depends on balancing diagnostic and therapeutic benefits with its potential toxicity. There are several approaches to minimizing the toxicity of Congo red that may allow its safer use in clinical trials. The key is to accurately determine the allowable dose and the route of administration. In this context, advanced pharmacokinetic models can help develop safe dosing protocols. Analysis of the half-life of Congo red in model organisms as well as its metabolism may enable the optimization of its use. Modern approaches such as nanoparticles can help deliver Congo red to specific sites in the body [52]. Such technologies can significantly reduce unwanted side effects while maintaining the diagnostic and therapeutic efficacy of Congo red. Moreover, the research highlights that Congo red is not a direct-acting mutagen. It is classified as a non-carcinogen in rat tests, which is attributed to its poor substrate efficiency for rat liver microsomal azo-reductase. Unlike certain benzidine-derived azo dyes that undergo efficient hepatic reduction and generate reactive metabolites linked to carcinogenicity, Congo red exhibits limited reduction by these liver enzymes [53]. Combinations with biomaterials (e.g., liposomes) may also be considered, which may provide more selective release of Congo red in the desired area of the particular organs [54]. Implementation of real-time toxicity monitoring strategies, including regular testing of biomarkers of organ damage (e.g., liver function tests, renal function tests), may help to quickly identify any adverse effects. In addition, the use of adjuvant therapies, such as heavy metal chelation or antioxidant therapy, may support the body’s detoxification process and reduce the risk of chronic damage. Congo red and its analogues offer unique possibilities to target the misfolded protein [50,55], preventing its further accumulation and blocking its toxicity in vitro [55]. Moreover, they show an ameliorative effect in vivo [55]. Congo red significantly prolongs the incubation period in scrapie-infected hamsters [56] and prevents the formation of and removes already formed polyglutamine aggregates in the R62 mouse model of Huntington’s disease [55]. This advantageous feature continues to make this azo dye an interesting prototype substance in the search for a therapeutic approach to several diseases.

Congo red may also play a role in the nanotheranostics of neurodegenerative diseases such as Alzheimer’s disease (AD) [52]. As studies show, Congo red can specifically detect amyloid plaques and form a biocompatible nanotheranostic system based on iron oxide magnetic nanoparticles with ultrasmall sizes and excellent magnetic properties. These properties enable magnetic resonance imaging of amyloid plaques and the targeted delivery and controlled release of H_2_O_2_ of therapeutic agents used in AD [52]. It should be emphasized that the feasibility of using Congo red/Rutin-MNPs in AD therapeutic applications in vitro and the effect of Congo red/Rutin-MNPs on the cytotoxicity of Aβ aggregation using SH-SY5Y neuroblastoma cells were also addressed by this study [52]. The study revealed that cell viability also increased to approximately 95% in the presence of Congo red/Rutin-MNPs, indicating that the delivery system had better protective effects on the cytotoxicity induced by Aβ in SH-SY5Y cells. In fact, a study conducted before in vivo imaging and subsequent therapeutic investigation clearly demonstrated that the nanotheranostics of Congo red/Rutin-MNPs [52], which can specifically detect amyloid plaques by magnetic resonance imaging, can effectively provide the targeted delivery of therapeutic agents in this AD model due to fully controlled drug release via a mechanism involving H_2_O_2_ response and the prevention of oxidative stress. Both in vitro and in vivo experiments were conducted to substantiate this possibility and to prove the remarkable disease-modifying effects of nanotheranostics. More importantly, intravenous administration of Congo red/Rutin-MNP was superior for detecting multiple amyloid plaques, sparing memory deficits and improving neurological changes in the brains of APPswe/PS1dE9 transgenic mice. As nanotheranostics, Congo red/Rutin-MNPs have demonstrated clinical utility and could provide more efficient and safer theranostic systems for AD [52].

Here, we provide evidence that the combination of CR-BLU-258 inhibited the growth and migration or promoted apoptosis of pancreatic cancer cells. Importantly, we showed that Congo red itself not only failed to significantly affect the survival of cancer cells, but also did not increase the percentage of apoptotic cells in normal human gastric fibroblasts. Even at the highest concentrations of Congo red used (60 µM, 100 µM), the percentage of apoptotic cells did not exceed 5%. Moreover, it was not detrimental to cell survival or LDH secretion in both the normal fibroblasts and the pancreatic cancer cells. We observed the higher IC_50_ value for CR-BLU-258 compared with BLU-258 alone, possibly due to several factors, including pharmacokinetic properties and mechanisms related to the complex interaction of both compounds. For instance, it is possible that CR-BLU-258 exhibits altered bioavailability or binding kinetics to its molecular target compared with BLU-258 alone. Co-aggregation with Congo red may affect cell penetration, stability, or affinity to target kinase(s), which may result in an apparently higher IC_50_ value. Changes in drug solubility or stability may affect how effectively the compound interacts with its molecular target. However, an important aspect of our study is that in the three-dimensional cellular model, the IC_50_ value for CR-BLU-258 was significantly lower than that of BLU-258 alone (Table 3). This indicates that the therapeutic effect of CR-BLU-258 is more pronounced under conditions that better mimic the tumor microenvironment, underscoring its clinical potential. The 3D models more closely reflect the conditions in solid tumors, including oxygen gradients, nutrient availability, and cell–cell interactions, suggesting that CR-BLU-258 may be more effective in vivo than simple IC50 measurements in conventional 2D models indicate. Finally, a higher IC_50_ seems to not necessarily indicate a lower therapeutic efficacy, especially if the CR-BLU-258 complex offers additional biological benefits such as prolonged activity, greater selectivity for tumor cells, or a more favorable toxicological profile.

In agreement with our previous studies, Congo red alone failed to affect the survival of normal bladder cells and exhibited an ability to form aggregates with sorafenib, not only resulting in inhibition of proliferation of these cells, but also showing reduced cytotoxicity to normal urothelial cells [21].

This opens new perspectives and possibilities for the combination of Congo red with sorafenib for cancer treatment, because of this adduct minimizing side effects on healthy tissue. Furthermore, these sorafenib–Congo red aggregates showed the ability to inhibit the migration and invasion of cancer cells, indicating their potential to prevent metastasis, one of the most dangerous aspects and consequences of cancer. For these reasons, further studies of tyrosine kinase inhibitors, showing potent antitumor activity in combination with supramolecular compounds, may open a new avenue for targeted therapy. Of note, other PDGFR and c-KIT inhibitors may also have potent antitumor activity in pancreatic cancer [11,12,57,58,59,60,61,62,63]. Taeger et al. demonstrated that dovitinib (TKI-258) can inhibit the growth of pancreatic cancer cells and reduce their motility [12]. In another study in vivo, TKI-258 led to a dose-dependent inhibition of tumor growth and reduced metastasis [12]. Studies by Yasuda et al. showed inhibition of proliferation and invasion in c-KIT-positive pancreatic cancer cell lines (PANC-1 and SW1990) under the influence of imatinib mesylate (STI-571) [11]. Our preliminary studies to date indicated that imatinib (STI-571) can form strong, stable complexes with CR (unpublished data). It is possible that such complexes would present much stronger and beneficial antitumor effects in pancreatic cancer than treatment with STI-571 alone.

In summary, our studies demonstrate for the first time the interaction between CR and BLU-258, as well as the effect of these compounds’ combination on pancreatic cancer cells. The obtained results provide convincing evidence that both the tested compounds, BLU-258 and CR-BLU-258, may be effective in the treatment of pancreatic cancer and deserve attention for a further justification of these compounds’ efficacy in in vivo conditions, which will be our goal in future studies.

## 4. Materials and Methods

### 4.1. Molecular Modeling

The interaction studies between avapritinib (BLU-258, AV) and Congo red (CR) molecules were performed using the Amorphous Cell module of BIOVIA Materials Studio 2020 software suite (BIOVIA Materials Studio 2020 software package; https://www.3ds.com/products-services/biovia/products/molecular-modeling-simulation/biovia-materials-studio/, accessed on 1 June 2024). The Amorphous Cell module includes a comprehensive collection of tools for creating three-dimensional periodic structures and constructing molecules in a cell using Monte Carlo methods, reducing tight interactions between atoms while guaranteeing a realistic distribution of torsion angles for any given forcefield. Structures can be built with any number of components at a specific density.

The Forcite Plus module has been used to optimize the generated structures, including the optimization of unit cell dimensions, applying ultra-fine quality settings of geometry optimization and smart algorithms. The convergence tolerance values were set to 2 × 10^−5^ kcal/mol for energy, 1 × 10^−3^ GPa for stress, 1 × 10^−3^ kcal/(Å × mol) for force, and 1 × 10^−5^ Å for displacement, with 5 × 10^4^ maximum iterations. All calculations were performed using the Condensed-phase Optimized Molecular Potentials for Atomistic Simulation Studies (COMPASS) III forcefield. As charge assignments are one of the characteristics provided in this forcefield specification, the charges were allocated using the COMPASS III forcefield. The electrostatic and van der Waals summation methods were both atoms-based with cubic spline truncation of non-bond energy terms, 18.5 Å cutoff distance, 1 Å spline width, and 0.5 Å buffer width.

The compositions of the studied models are presented in Table 4.

### 4.2. Sample Preparation

A 5 mM Congo red solution (TCI America, Portland, OR, USA) in PBS (pH 7.4; EURx, Gdansk, Poland) was heated to 100 °C for 2 min, and then slowly cooled to room temperature over approximately 10 min, followed by dilution with PBS. Avapritinib (BLU-285, AV, MedChem Express, Monmouth Junction, NJ, USA) was dissolved in 10 mM DMSO (Sigma Aldrich, St. Louis, MO, USA), then pre-diluted with pure water to a concentration of 1 mM. Subsequently, this solution was mixed with the previously prepared diluted CR to achieve the required concentrations as specified in the text.

### 4.3. Dynamic Light Scattering (DLS)

Dynamic light scattering (DLS) measurements were carried out at 25 °C using a Zetasizer Nano ZSP equipped with a 633 nm red laser (Malvern Instruments Ltd., Worcestershire, UK). The measurements were performed using a plastic cuvette (BRAND^®^ UV cuvette micro, BR759200) (Sigma-Aldrich, St. Louis, MO, USA). Data were analyzed with Malvern Zetasizer 7.13 software (Malvern Instruments Ltd., Worcestershire, UK). Particle size distribution (PSD) numbers were used for reporting sizes. Each sample underwent three DLS measurements, with each measurement consisting of ten runs of 30 s each. A detection angle of 173° was employed for the size determination.

### 4.4. UV-Vis Spectroscopy

UV-Vis spectra were recorded using a Cary 300 spectrophotometer (CaryWinUV, Perlan, Agilent Technologies, Inc., Santa Clara, CA, USA). Measurements were performed in scan mode over a wavelength range of 250 nm to 600 nm, with a scan speed of 200 nm/min and a slit width of 3 nm.

### 4.5. Cell Line and Cell Culture

Human pancreatic cell lines PANC-1 and BxPC-3 were obtained from the American Type Culture Collection (ATCC, Manassas, VA, USA). Pancreatic cells were cultured in DMEM (PANC-1 cell lines) and RPMI-1640 medium (BxPC-3 cell lines) supplemented with 10% fetal bovine serum (FBS) and 1% penicillin/streptomycin (Biowest, Riverside, MO, USA) at 37 °C, 5% CO_2_, and 95% humidity [64]. The pancreatic cells were passaged two or three times a week.

Human gastric fibroblasts (HGFs) were purchased from Innoprot^®^(Innoprot, Bizkaia, Spain) and maintained in Innoprot-recommended media and conditions. The media were changed every two days. The HGF cells were passaged two times a week.

### 4.6. Treatments of the Cells

Avapritinib (BLU-258, MedChem Express, Monmouth Junction, NJ, USA) was dissolved in DMSO with a stock concentration of 10 mM, and the stock solution was stored at –20 °C. BLU-258 was freshly diluted to the predetermined concentrations with culture medium. Congo red (5 mM) was diluted with PBS (pH 7.4), heated to 100 °C for 2 min, and slowly cooled to ambient temperature for approximately 10 min. Avapritinib with diluted CR was prepared earlier to achieve the appropriate concentrations described in the text. To exclude the effect of solvent, DMSO was added to control cells. All samples contained a final concentration of 0.1% DMSO; vehicle control samples were treated with cell culture media containing 0.1% DMSO.

### 4.7. Cell Metabolic Viability Assays

Pancreatic cell lines were seeded on 96-well plates at a density of 5 × 10^3^ cells/well. After 24-h growth at 37 °C in a humidified atmosphere, the culture medium was removed, and BLU-258 (in concentration range of 0.25–100 µM) or analyzed aggregates with Congo red (molarity ratio 5:1) or CR (in concentration range of 1–100 µM) were added to the pancreatic cells. The experiments were carried out for 24, 48, and 72 h at 37 °C in a humidified atmosphere. The MTS (Promega, Madison, WI, USA) [65] was used to determine the compounds’ influence on viability of pancreatic cancer cells (PANC-1 and BxPC3). An Epoch Microplate Spectrophotometer (BioTek Instruments Inc., Winooski, VT, USA) was used to measure the absorbance at 570 nm. The effects of BLU-258 or CR-BLU-258 aggregates were presented as proliferation curves.

Equation for the cell viability:

Cell viability = (Ai − A0)/(Ak − A0) × 100%

where

- Ai is the average absorbance after incubation with the tested compounds,
- A0 is the average absorbance of the control at the beginning of the experiment,
- Ak is the average absorbance of the control after 48 h of incubation without compounds.

### 4.8. Cell Cytotoxicity by LDH Assay

After 48 h of culturing the PANC-1, BxPC3, and HGF cell lines, the medium supernatant was collected to evaluate the cytotoxicity of the investigated compounds by lactate dehydrogenase (LDH, Thermo Fisher Scientific, Waltham, MA, USA) assay.

The LDH assay was performed according to the manufacturer’s protocol as previously described [66].

### 4.9. Cell Survival Analysis by Flow Cytometry

A PE Annexin V Apoptosis Detection Kit I (BD Biosciences, San Jose, CA, USA) was used following the manufacturer’s instructions. Samples were analyzed by flow cytometry on a BD FACS Canto^TM^ instrument (Becton Dickinson, New York, NY, USA). Apoptotic cells were defined as Annexin V-positive cells in the test.

### 4.10. Migration Assay

Confluent pancreatic cells were cultured in DMEM (PANC-1 cell lines) and RPMI-1640 (BxPC-3 cell lines) media with 0.5% BSA for 24 h. Next, cells were treated with BLU-258 or CR-BLU-258 (IC_10_ concentration) for 24 h. Control cells were cultured under identical conditions, without investigated compounds, with 0.1% DMSO. A perpendicular scratch wound was generated by scratching with a pipette tip and washed with phosphate buffered saline (PBS) to remove floating cells. After 24 h of incubation, scratches were monitored and photographed using a light microscope (AxioVert A1, Carl Zeiss, Oberkochen, Germany) with automatic scanning table and ZEN Pro 2.3 software (Carl Zeiss, Oberkochen, Germany). The images were analyzed using FiJi Image J 1.54f software (National Institute of Health, Bethesda, MD, USA), as previously described [67].

### 4.11. Western Blot Analysis

The PANC-1 and BxPC3 cell lines were seeded on 6-well plates in the 80% confluence and were serum-deprived for 12 h before stimulation. Cells were exposed to BLU-258 (IC_50_ concentration) and analyzed aggregates with Congo red for 3 or 6 h. The control plates were cells cultured in a standard medium without the investigated compounds, with 0.1% DMSO.

Western blot analysis (cell lysis, the list of primary antibodies used to detect the investigated proteins, Western blot detection, and visualization of blots) was performed in the same manner as previously described for sorafenib [21].

The list of primary and secondary antibodies used is given in Table 5.

### 4.12. Tumor Spheroid Generation

LifeGel Digestion Kit (cat. no. L912131411) and LifeGel 3D cell culture plates (Real Research, Krakow, Poland) were used to form tumor spheroids. LifeGel 3D cell culture plates were pre-equilibrated at 37 °C, which was gassed with 5% CO_2_. The medium was removed from the LifeGel layer before cells were seeded in 200 μL of culture medium per well, and the plates were returned to the humidified incubator. The same culture medium was used for 3D as for 2D for each cell line. The 3D cell cultures were seeded with 2 × 10^3^ PANC-1 or BxPC-3 cells per well. The morphology of 3D cell cultures was monitored using a Zeiss Telaval 31 microscope, 50× magnification, bright field 3D images (Carl Zeiss, Oberkochen, Germany).

The 3D cell cultures were cultured for 5 days without changing medium.

### 4.13. CTB Analysis

After 5 days in 3D cell cultures, the culture medium was removed, and then BLU-258 (in the concentration range of 0.5–250 µM), analyzed aggregates with Congo red (molar ratio 5:1), or appropriate fresh medium with 0.1% DMSO (control) was added. Experiments were carried out for 48 h at 37 °C in a humidified atmosphere.

The metabolic activity of the spheroids was monitored using the CTB analysis (CellTiter-Blue^®^ Cell Viability Assay, Promega, Madison, WI, USA) according to the manufacturer’s instructions. Briefly, on the day of the experiment, 20 µL of CellTiter-Blue^®^ reagent was added to each well and incubated for 4 h in culturing conditions. The fluorescence intensity was measured at 560 nm excitation and 590 nm emission using the Victor Nivo Multimode Plate Reader (PerkinElmer^®^ Inc., Waltham, MA, EUA).

### 4.14. Statistical Analysis

Unless stated otherwise, the data are expressed as the mean ± standard deviation (SD). Statistical analysis was performed by one-way ANOVA with Dunnett’s post-test, Mann–Whitney U test, or Student’s *t*-test using GraphPad 5.01 software. Differences with a value of *p* < 0.05 were considered statistically significant. The values of IC_50_ and IC_90_ were obtained by fitting a sigmoidal model to the data showing the inhibitor curves for BLU-258 or CR-BLU-258 using Origin 2024.

## 5. Conclusions

Our studies have shown that Congo red forms stable, heterogeneous aggregates with BLU-258. Furthermore, our studies have allowed us to compare the effects of BLU-258 alone or as CR-BLU-258. We have shown that the tested compounds, BLU-258 and CR-BLU-258, had a cytostatic and cytotoxic effect on pancreatic cancer cells (PANC-1 and BxPC3 lines), reduced their migration, and affected the level of phosphorylation of the Akt and Erk1/2 proteins. In addition, our studies have revealed an increased sensitivity of 2D pancreatic cell cultures to BLU-258 and CR-BLU-258. We have shown an increased sensitivity of 3D cell cultures to CR-BLU-258 compared with the inhibitor alone. This may be because BLU-258 itself forms large aggregates, the bioavailability of which is limited due to their size and solubility, as reflected by DLS measurements. Importantly, our studies also showed that the tested CR-BLU-258 complex exhibits a significantly lower IC_50_ value compared with the inhibitor alone. Further studies of co-aggregates formed by combining supramolecular compounds with tyrosine kinase inhibitors may open new possibilities for more effective and safer anticancer therapies.

## Figures and Tables

**Table 1:** Intermolecular interaction energies and volumes of the unit cells of the studied systems. Values in the lowest row have been calculated according to the formula AV50CR50–0.5 (AV100 + CR100).

Model	Intermolecular Interactions Energy per One Molecule (kcal/mol)	Volume (A3)
AV100	−44.39	125,196
CR100	−13.32	103,220
AV50CR50	−36.92	116,772
AV50CR50–0.5 (AV100 + CR100)	−8.06	2564

**Table 2:** Values of IC_10_, IC_50_, and IC_90_ obtained by fitting a sigmoidal model to the data showing the inhibitor curves for BLU-285 and CR-BLU-258. Data are expressed as mean ± standard error.

Cell Line	BLU-258 (μM)			CR-BLU-258 (μM)		
	IC_10_	IC_50_	IC_90_	IC_10_	IC_50_	IC_90_
PANC-1	1.69 ± 0.34	3.97 ± 0.11	8.27 ± 0.31	1.46 ± 0.2	4.05 ± 0.12	7.27 ± 0.31
BxPC3	0.23 ± 0.03	1.74 ± 0.07	9.6 ± 0.16	0.23 ± 0.03	2.24 ± 0.07	10.45 ± 0.18

**Table 3:** Values of IC_10_ and IC_50_ for 3D cell cultures obtained by fitting a sigmoidal model to the data, showing the inhibitor curves for BLU-285 and CR-BLU-258. Data are expressed as mean ± standard error.

3D Cell Culture	BLU-258 (μM)		CR-BLU-258 (μM)	
	IC_10_	IC_50_	IC_10_	IC_50_
PANC-1	0.63 ± 0.12	165.73 ± 0.19	0.38 ± 0.13	36.89 ± 0.10
BxPC3	5.13 ± 0.13	>250	3.37 ± 0.14	18.86 ± 0.14

**Table 4:** Composition of the studied models.

Model	Composition
Congo red (100CR)	100 Congo red molecules
BLU-258 (100AV)	100 BLU-258 molecules
BLU-258–Congo red (50AV50CR)	50 BLU-258 molecules and 50 Congo red molecules

**Table 5:** List of the antibodies and conditions that were used in this study.

Antibody	Material Number	Host Species	Dilution	Vendor
Akt	#9272	rabbit	1:1000	Cell Signaling Technology Inc. (Danvers, MA, USA)
phospho-AKT (Ser473)	#4060	rabbit	1:1000	
p44/42 MAPK (ERK1/2)	#4695	rabbit	1:1000	
phospho-p44/42 MAPK (Thr202/Tyr204) (phospho-ERK1/2)	#9106	mouse	1:1000	
Hsp90	610418	mouse	1:4000	BD Transduction Laboratories
Anti-rabbit IgG, HRP-linked antibody	#7074	rabbit	1:3000	Cell Signaling Technology Inc. (Danvers, MA, USA)
Anti-mouse IgG, HRP-linked antibody	#7076	mouse	1:3000	Cell Signaling Technology Inc. (Danvers, MA, USA)

## Data Availability

The datasets used and/or analyzed during the current study are available from the corresponding author on reasonable request.

## Supplementary Materials

The following supporting information can be downloaded at: https://www.mdpi.com/article/10.3390/ijms26051980/s1.
