# Supplementary material for: Interaction of Avapritinib with Congo Red in Pancreatic Cancer Cells: Molecular Modeling and Biophysical Studies
Source: Int J Mol Sci. 2025 Feb 25;26(5):1980. doi: 10.3390/ijms26051980 (PMC11901030; doi:10.3390/ijms26051980)
Supplement: Supplementary file 1 [file ijms-26-01980-s001.zip › ijms-3454518-supplementary.pdf]

Interaction of Avapritinib with Congo Red in Pancreatic Cancer Cells: Molecular Modeling and Biophysical Studies

Malgorzata Lasota, Daniel Jankowski, Anna Wisniewska, Lukasz Szeleszczuk, Anna Misterka-Kozaka, Marta Kaczor-Kaminska, Marta Zarzycka, Maksym Patena, Tomasz Brzozowski

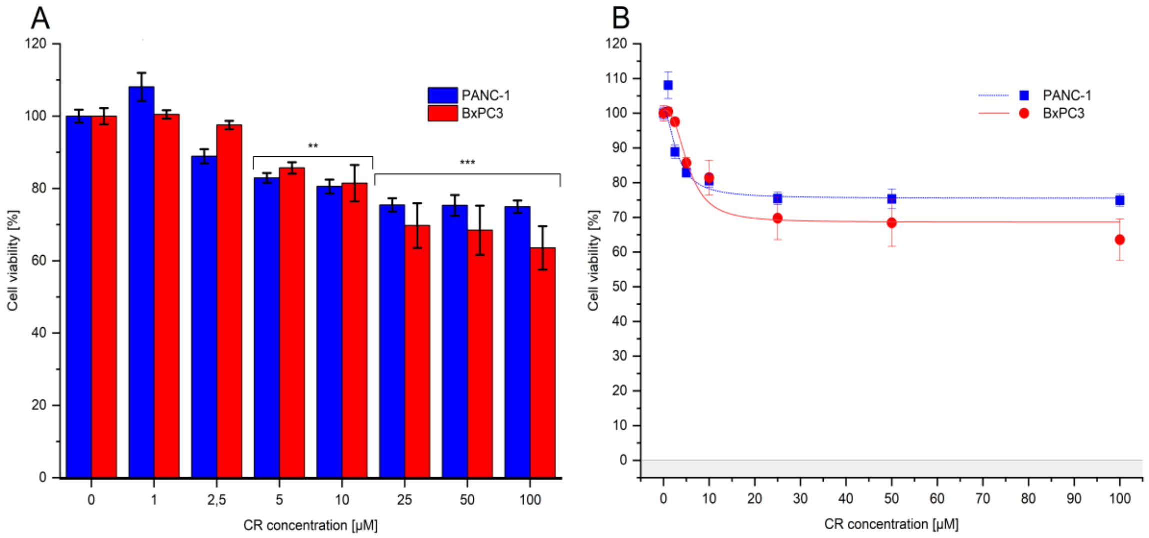

Supplementary Figure S1. The dose-dependent effect of Congo red (CR) on the viability of PANC-1 and BxPC3 cells after 48 h incubation (A, B). Statistical significance between untreated and treated samples was evaluated using ANNOVA with Dunnett post-test: ns – non-significant ($p > 0.05$) in comparison with a control sample (without CR); ** $0.001 < p < 0.01$; *** $p < 0.001$.

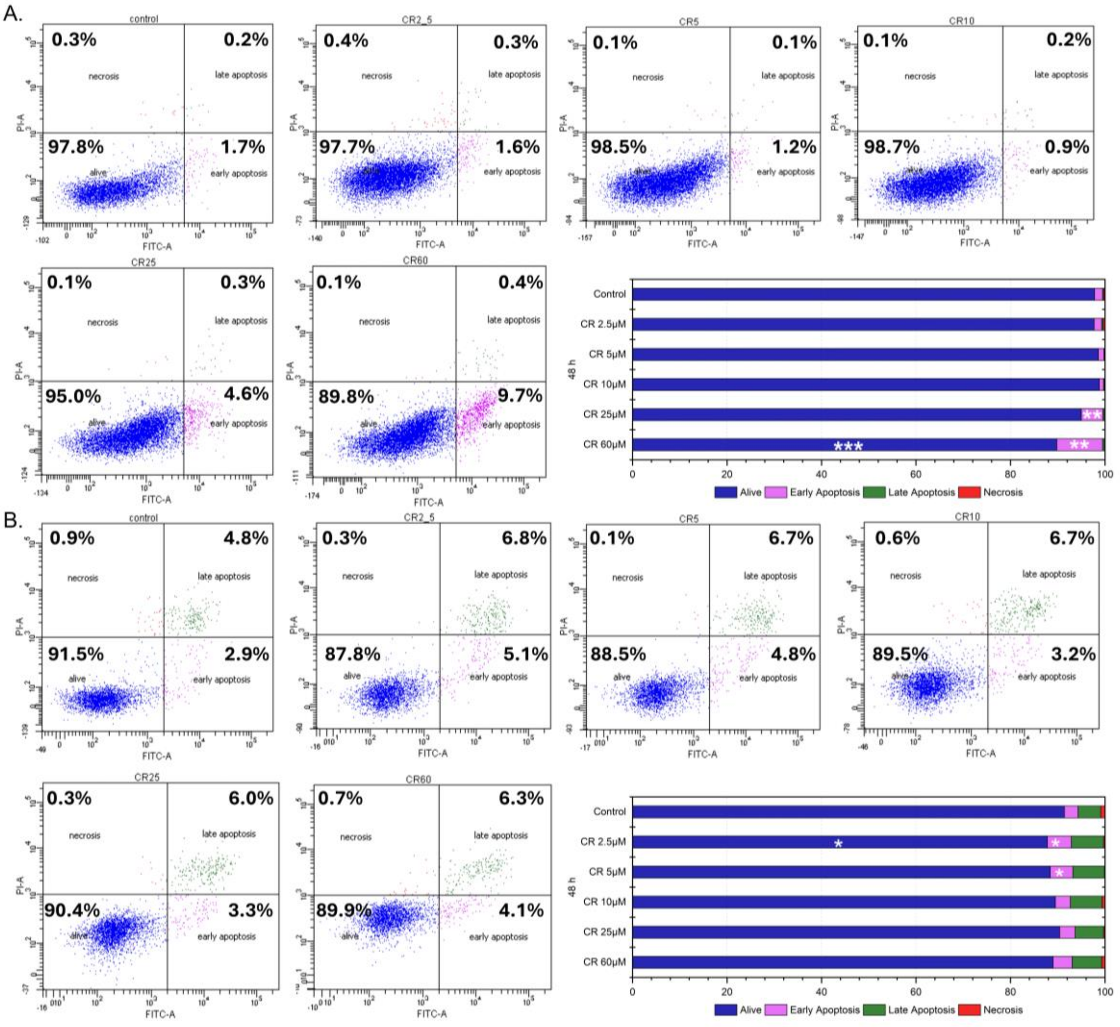

Supplementary Figure S2. The effect of Congo red (CR) on apoptosis and necrosis of pancreatic cells. The images show flow cytometry analysis of Annexin V and PI staining presented on a dot plot graph. Graphic representation of four cell states: alive - the lower left square; cells undergoing necrosis - the upper left square; cells in early apoptosis - the right lower square; and cells in late apoptosis the upper right square. PANC-1 (A) and BxPC-3 (B) cells were incubated with CR; a the concentrations range 0 - 60μM). Cumulative bar charts show the interrelation between the state of cells after 48 h to CR on PANC-1 (A) and BxPC3 (B) cells. Statistical significance between the investigated samples was evaluated using U Mann-Whitney's test: ns – non-significant ($p > 0.05$), * $0.01 < p < 0.05$, ** $0.001 < p < 0.01$).

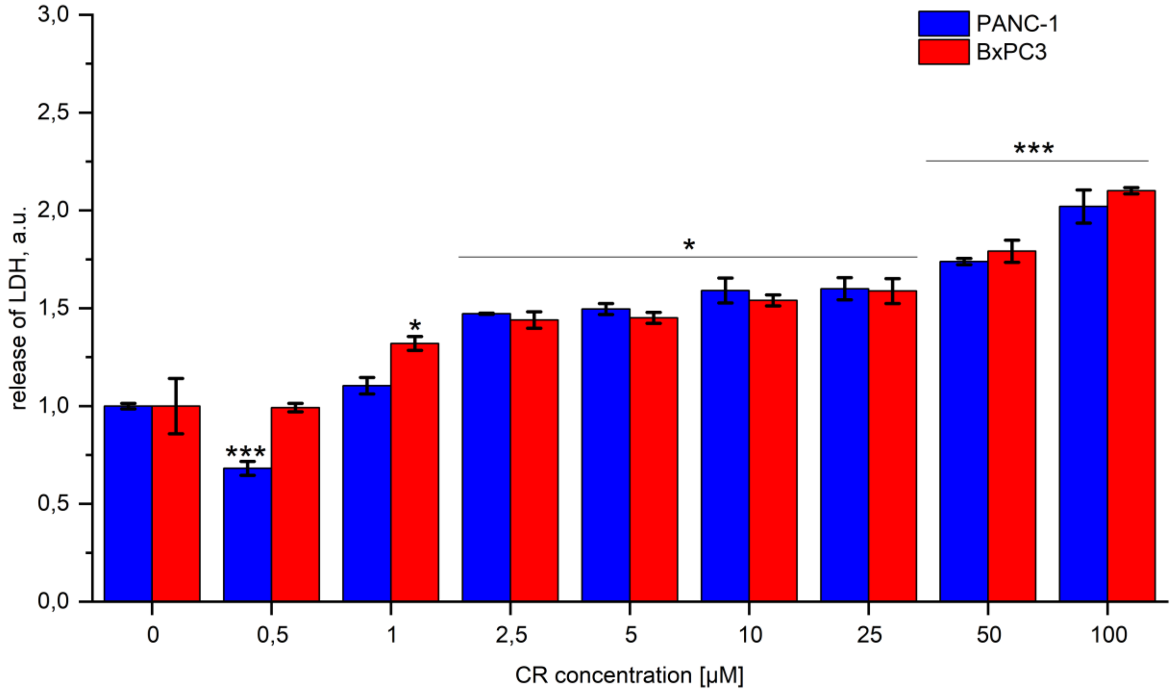

Supplementary Figure S3. Cytotoxic effect of Congo red (CR) on pancreatic cell lines was assessed using LDH assay after 48h. Statistical significance: between untreated and treated samples was assessed using ANOVA with Dunnett's posttest: ns - not significant ($p > 0.05$) compared to the control sample (without BLU-258 or CR-BLU-258; with 0.1% DMSO); * $0.01 < p < 0.05$; *** $p < 0.001$.

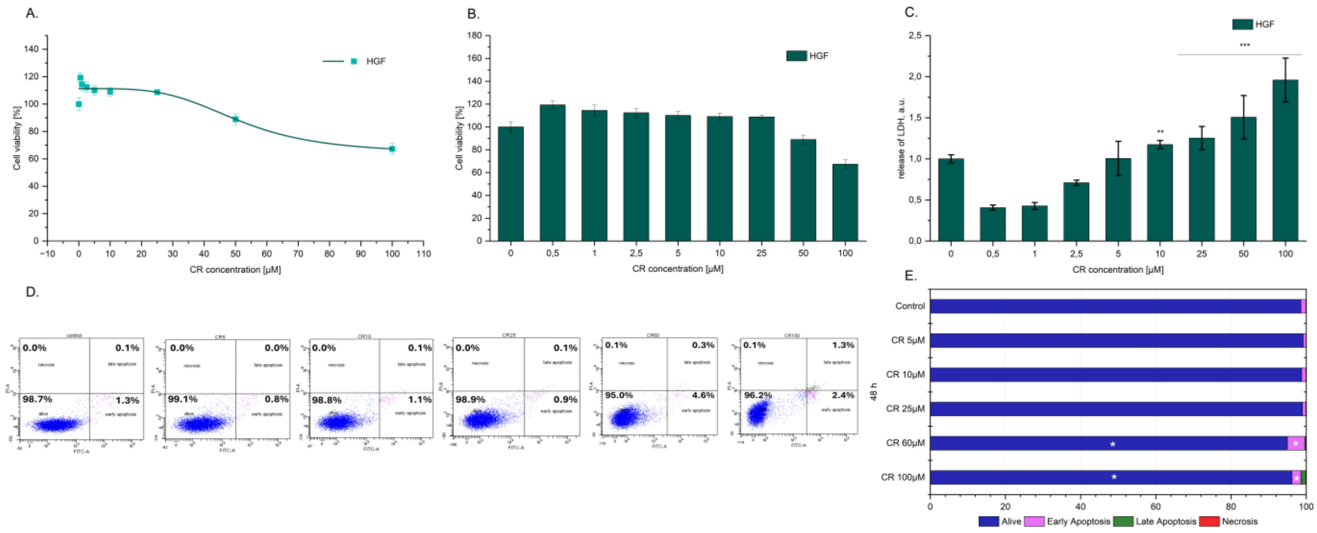

Supplementary Figure S4. Effect of Congo red (CR) on normal human gastric fibroblast (HGF) cells. A, B – Dose-dependent effect of Congo red on the viability of HGF cells after 48 h of incubation; C – Evaluation of the cytotoxicity of CR on HGF cells evaluated by the LDH assay. Statistical significance between untreated and treated samples was evaluated by ANNOVA with Dunnett's post-test: ns - not statistically significant ($p > 0.05$) compared to the control sample (without CR); * $0.01 < p < 0.05$, ** $0.001 < p < 0.01$, *** $p < 0.001$; D, E – Effect of Congo red on apoptosis and necrosis of human gastric fibroblast.

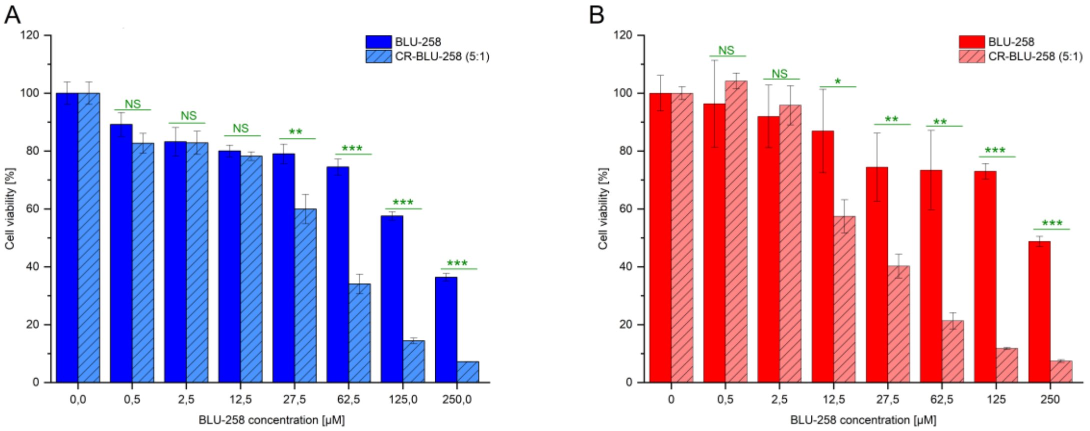

Supplementary Figure S5. The dose-dependent effect of BLU-258 (alone) and in aggregates with Congo red (CR-BLU-258) on the viability of 3D PANC-1 (A) and 3D BxPC3 (B) cells after 48 h incubation. Statistical significance between BLU-258 and CR-BLU258 was assessed using Student's t-test - green asterisks; NS – not significance ($p>0.05$), * $0.01 < p < 0.05$, ** $0.001 < p < 0.01$, *** $p < 0.001$.
